# Long-term clinical outcomes in patients with non-ST-segment Elevation Acute Coronary Syndrome and ST-segment elevation myocardial infarction with thrombolysis in myocardial infarction 0 flow

**DOI:** 10.1016/j.ijcha.2023.101254

**Published:** 2023-08-29

**Authors:** Bart R.A. Aarts, Frederik T.W. Groenland, Jaimy Elscot, Tara Neleman, Jeroen M. Wilschut, Isabella Kardys, Rutger-Jan Nuis, Roberto Diletti, Joost Daemen, Nicolas M. Van Mieghem, Wijnand K. den Dekker

**Affiliations:** Department of Cardiology, Erasmus MC Cardiovascular Institute, University Medical Center Rotterdam, The Netherlands

**Keywords:** Acute coronary syndrome, Percutaneous coronary intervention, TIMI 0 flow

## Abstract

**Background:**

Thrombolysis in Myocardial Infarction (TIMI) 0 flow often characterizes ST-segment Elevation Myocardial Infarction (STEMI) patients, but may also feature in non-ST-segment Elevation Acute Coronary Syndrome (NSTE-ACS). Since recanalization usually occurs later in NSTE-ACS patients, the aim of this study was to assess whether patients presenting with NSTE-ACS and TIMI 0 flow have worse clinical outcomes as compared to patients presenting with STEMI and TIMI 0 flow.

**Methods:**

A single-center retrospective cohort study was conducted with patients treated for NSTE-ACS and STEMI with TIMI 0 flow at diagnostic angiogram between January 2015 and December 2019. The two patient groups were 1:1 matched using a propensity score logistic regression model. The primary outcome was Major Adverse Cardiac Events (MACE), a composite of all-cause mortality, any myocardial infarction, coronary artery bypass graft, urgent target vessel revascularization or stroke during long term follow-up.

**Results:**

The total population consisted of 1255 ACS patients, of which 249 NSTE-ACS and 1006 STEMI patients. After propensity score matching, 234 NSTE-ACS patients were matched with 234 STEMI patients. In this matched population, the mean age was 62.6 (±12.4) years and 75.2 % of the patients was male. The median follow-up time was 3.2 years. MACE rates during follow-up were similar between the two matched groups (HR = 0.84 [95 % CI 0.60 – 1.12] with p = 0.33) with cumulative event-free survival of 63.3 % in the NSTE-ACS group vs 59.3 % in the STEMI group at 6 year follow-up.

**Conclusion:**

In this retrospective study, a culprit lesion with TIMI 0 flow has similar clinical outcome in NSTE-ACS and STEMI patients. Further research is warranted to determine optimal the timing of PCI in NSTE-ACS patients with TIMI 0 flow.

## 1 Introduction

Thrombolysis in Myocardial Infarction (TIMI) 0 flow is defined as a totally occluded vessel with no antegrade flow beyond the point of occlusion [1]. TIMI 0/1 flow is most common in ST-segment Elevation Myocardial Infarction (STEMI) patients [2]. TIMI 0 flow may also feature in approximately one third of patients with non-ST-segment Elevation Acute Coronary Syndrome (NSTE-ACS, including non-ST-segment Elevation Myocardial Infarction (NSTEMI) and Unstable Angina) [3], [4]. However, in contrast to STEMI patients, this is not perceptible on the electrocardiogram as ST-segment elevation.

TIMI 0 flow at the diagnostic angiogram is known to be associated with worse outcomes in STEMI patients [2], [5], [6], [7], [8], [9]. Therefore, rapid recanalization to restore blood flow is mandatory to improve outcome, which is also stated in the guidelines [10]. During the study period, the 2015 NSTE-ACS guidelines recommended an invasive strategy (i.e. coronary angiography) within 24 to 72 h after developing symptoms [11]. Two recent meta-analyses showed inconclusive results with regards to rapid versus delayed reperfusion in NSTE-ACS patient outcomes [12], [13]. In the 2015 guidelines, early (<24 h) coronary angiography was exclusively recommended in high risk NSTE-ACS patients [11] to decrease risk of death, myocardial infarction (MI) or stroke [14]. However, patients with NSTE-ACS and TIMI 0 flow might present without high risk features. Therefore, these NSTE-ACS patients might be excluded from early emergent coronary angiography, in spite of having a totally occluded coronary artery. As a result, recanalization often occurs later than in patients with STEMI and TIMI 0 flow.

The aim of this study was to assess whether patients with NSTE-ACS and TIMI 0 flow have worse outcomes compared to patients with STEMI and TIMI 0 flow.

## 2 Methods

### 2.1 Study design and patient population

A single-center, retrospective cohort study was conducted with acute coronary syndrome (ACS) patients undergoing percutaneous coronary intervention (PCI) between 01 and 01-2015 and 31-12-2019 in the Erasmus University Medical Center, Rotterdam, the Netherlands. Patients with a history of coronary artery bypass graft (CABG), heart transplant (HTx), TIMI 0 flow due to chronically total occlusion (CTO), no TIMI 0 flow or no available medical data were excluded. The Medical Ethics Committee of the Erasmus Medical Center waived approval due to the retrospective nature of the data collection.

### 2.2 Baseline and procedural characteristics assessment

ACS patients were divided into two groups: NSTE-ACS patients and STEMI patients. For NSTE–ACS patients, the Global Registry of Acute Coronary Events (GRACE) score was extracted from the patients’ medical records. If the GRACE score was not available, it was calculated using the variables age, heart rate, systolic blood pressure, creatinin levels, cardiac arrest at admission, ST-segment deviation on the ECG, abnormal cardiac enzymes and the Killip class at presentation [15]. Timing of PCI was computed using the patient reported onset of explicit ACS symptoms (when available) and the start time of the procedure.

Electrocardiograms were reviewed and classified using the fourth universal definition for myocardial infarction, defined as the presence of acute myocardial injury detected by abnormal cardiac biomarkers in the setting of evidence of acute myocardial ischemia [16]. The TIMI flow, vessel lesion classification and the presence of collaterals were assessed using the coronary angiograms. For the evaluation of collaterals, the RENTROP classification was used [17]. Any disputes were resolved mutually (WD and BA).

### 2.3 Study outcomes

The primary endpoint was defined as long-term Major Adverse Cardiac Event (MACE), a composite of all-cause mortality, any MI, CABG, urgent target vessel revascularization (UTVR) or stroke, whichever occurred first. MI was diagnosed using the fourth universal definition of MI (16). Stroke was diagnosed using the American Heart Association/American Stroke Association (AHA/ASA) guidelines [18]. UTVR was defined as a repeat PCI in the index coronary artery. Secondary endpoints consisted of the individual components of MACE.

### 2.4 Data collection

Baseline characteristics, procedural characteristics and follow-up data were obtained via the hospital’s electronic medical records. Data on all-cause mortality was extracted from the municipal civil registry. When not available in the hospital’s records, follow-up data were collected through a written patient questionnaire or a telephone survey. Only when a patient had given their explicit consent in the past, the general practitioner was contacted and asked for missing follow-up data.

### 2.5 Statistical analysis

Categorical variables are expressed as percentages (%) and compared using either the Pearson’s Chi-squared test, or the Fisher’s Exact test, as appropriate. Continuous variables are presented as means ± standard deviation or median and 25th-75th percentiles. For comparison of continuous variables, the unpaired *t*-test and Mann-Whitney *U* test was used, depending on variable distribution. The difference in outcome event rates between NSTE-ACS and STEMI patients was assessed by time-to-event analyses using the Kaplan-Meier method and the log-rank test. Follow-up of the patients lasted until the study follow-up end date (June 1st, 2022), until the time of event or until patients were lost to follow-up, at which moment patients were censored.

To account for differences in the baseline characteristics between the two groups, a propensity score (PS) matching analysis was performed. The following covariates, all of which are known prognostic factors in ACS patients, were chosen to construct the propensity score: age, gender, history of PCI, history of MI, history of peripheral artery disease, history of stroke, history of chronic kidney disease, history of hypertension, history of hypercholesterolemia, family history, history of diabetes, current smoker and the culprit vessel. The matching was performed without replacement and with a specified caliper of 0.1. SPSS was used, with the *fuzzy* extension. Because in a matched population the patients cannot be considered as independent [19], the multivariable analysis of the time-to-event MACE outcomes was performed using Cox models with robust standard errors in *R*.

The statistical analyses were performed using IBM SPSS statistics version 26 and *R*studio version 1.4.1106. Results were considered significant at a 2-tailed P-value < 0.05.

### 2.6 Patient selection

**Fig. 1:**
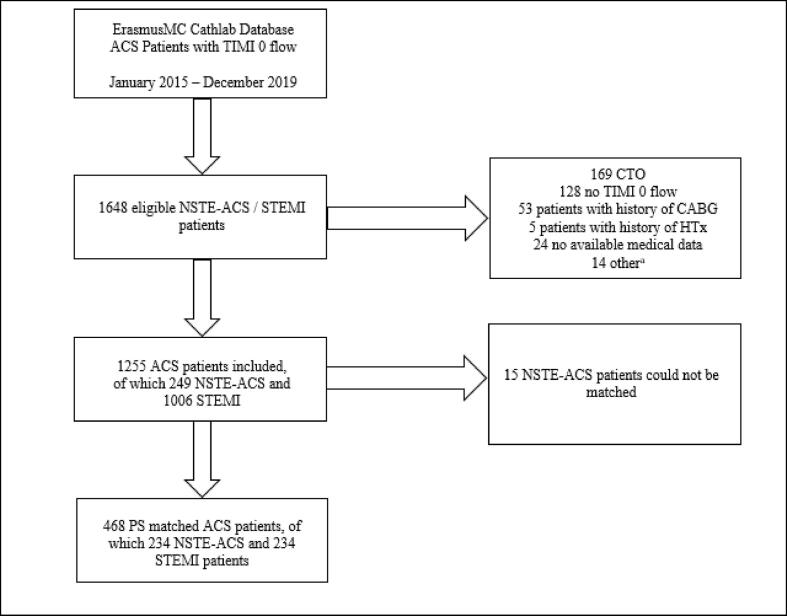
Flowchart patient selection ^a^Other = 3 post-operation complications, 2 coronary angiography (CAG) performed as work-up to *trans*-aortic valve implantation, 1 CAG performed as work-up to lung transplantation, 5 iatrogenic dissections, 1 CAG performed for heart failure, 1 deceased patient due to wrongfully intubation, 1 patient with solely coronary spams TIMI flow = Thrombolysis In Myocardial Infarction Flow; ACS = Acute Coronary Syndrome; NSTE-ACS = Non-ST-Segment Elevated ACS; STEMI = ST-segment Elevated Myocardial Infarction; CTO = Chronic Total Occlusion; CABG = Coronary Artery Bypass Graft; HTx = Heart Transplantation, PS = Propensity Score.

In Fig. 1, a detailed explanation of the patient selection and exclusion is shown. Propensity score matching was performed with 249 NSTE-ACS patients, using a propensity score based on the aforementioned covariates. The model’s reliability was tested and confirmed using goodness-of fit (*C*-statistic 0.62, Hosmer-Lemeshow test p = 0.45). After PS Matching, 234 NSTE-ACS patients were matched with 234 STEMI patients. There were no exact matches and 15 NSTE-ACS patients could not be matched. Thus, a matched population of 468 ACS patients was formed.

As shown in Table 1, for the variables hypercholesterolemia and family history more than 5 % data was missing in the STEMI group (respectively 94.6 % and 93.8 % available data). In addition, <95 % data was available for the GRACE scores in the STEMI group (84.9 %). For the procedural characteristics, shown in Table 2, Table 3, complete data was available for both groups (except for time to procedure, which was missing in 13.7 % in the total population and 22.2 % in the matched population).

**Table 1:** Baseline Characteristics.

Variable	**Total**	**STEMI**	**NSTE-ACS**	**P-value**	**Total**	**STEMI**	**NSTE-ACS**	**P-value**	**Standardized mean difference**
	**(n=1255)**	**(n=1006)**	**(n=249)**		**(n=468)**	**(n=234)**	**(n=234)**		
Age (years)	63.2 [±12.7]	63.3 [±12.8]	62.9 [±12.0]	0.68	62.6 [±12.4]	62.4 [±12.8]	62.8 [±12.1]	0.74	0.03
Male	956/1255 (76.2)	766/1006 (76.1)	190/249 (76.3)	1.00	352/468 (75.2)	174/234 (74.4)	178/234 (76.1)	0.75	0.05
Hypertension	531/1215 (43.7)	403/971 (41.5)	128/244 (52.5)	**0.003**	221/468 (47.2)	97/234 (41.5)	124/234 (53.0)	**0.016**	0.24
Hypercholesterolemia	420/1194 (35.2)	312/952 (32.8)	108/242 (44.6)	**0.001**	180/468 (38.5)	78/234 (33.3)	102/234 (43.6)	**0.029**	0.23
Diabetes	180/1228 (14.7)	143/982 (14.6)	37/246 (15.0)	0.93	68/468 (14.5)	36/234 (15.4)	32/234 (13.7)	0.69	−0.03
Diet	13/171 (7.6)	10/136 (7.4)	3/35 (8.6)	1.00	4/66 (6.1)	2/36 (5.6)	2/30 (6.7)	1.00	0.05
Metformin	97/171 (56.7)	79/136 (58.1)	18/35 (51.4)	0.61	37/66 (56.1)	21/36 (58.3)	16/30 (53.3)	0.87	−0.01
Insulin	61/171 (35.7)	47/136 (34.6)	14/35 (40.0)	0.69	25/66 (37.9)	13/36 (36.1)	12/30 (40.0)	0.95	0.01
Family History	459/1182 (38.8)	361/944 (38.2)	98/238 (41.2)	0.45	179/468 (38.2)	83/234 (35.5)	96/234 (41.0)	0.25	0.11
Current Smoker (<1 year)	513/1218 (42.1)	417/972 (42.9)	96/246 (39.0)	0.30	205/468 (43.8)	115/234 (49.1)	90/234 (38.5)	**0.025**	−0.21
COPD	86/1246 (6.9)	67/1000 (6.7)	19/246 (7.7)	0.67	32/468 (6.8)	14/234 (6.0)	18/234 (7.7)	0.58	0.07
Previous MI	192/1248 (15.4)	138/1002 (13.8)	54/246 (22.0)	**0.002**	91/468 (19.4)	41/234 (17.5)	50/234 (21.4)	0.35	0.08
Previous PCI	201/1251 (16.1)	149/1004 (14.8)	52/247 (21.1)	**0.022**	90/468 (19.2)	43/234 (18.4)	47/234 (20.1)	0.73	0.04
Previous Stroke	83/1249 (6.6)	61/1003 (6.1)	22/246 (8.9)	0.14	35/468 (7.5)	14/234 (6.0)	21/234 (9.0)	0.29	0.13
Previous Peripheral Artery Disease	46/1247 (3.7)	35/1001 (3.5)	11/246 (4.5)	0.59	20/468 (4.3)	10/234 (4.3)	10/234 (4.3)	1.00	0.0
Chronic Kidney Disease	205/1232 (16.6)	171/986 (17.3)	34/246 (13.8)	0.22	67/468 (14.3)	36/234 (15.4)	31/234 (13.2)	0.60	−0.06
Creatinin (at presentation)	86 [74–103]^a^	87 [74–104]^a^	83 [72–98]^a^	**0.013**	84 [73–100]^a^	87 [74–104]^a^	83 [71–95]^a^	**0.018**	−0.05
eGFR (at presentation)	78 [64–91]^a^	77 [63–92]^a^	81 [66–91]^a^	0.10	80 [65–92]^a^	79 [64–93]^a^	82 [66–91]^a^	0.31	0.10
VF/VT before angiogram	142/1217 (11.7)	126/976 (12.9)	16/241 (6.6)	**0.009**	41/452 (9.1)	29/225 (12.9)	12/227 (5.3)	**0.008**	−0.33
Killip Class at Presentation				**0.002**				**<0.001**	
1	1163/1245 (93.4)	921/998 (92.3)	242/247 (98.0)		443/464 (95.5)	213/232 (91.8)	230/232 (99.1)		0.27
≥2	82/1245 (6.6)	77/998 (7.7)	5/247 (2.0)		21/464 (4.5)	19/232 (8.2)	2/232 (0.9)		−0.27
GRACE-score	115.29 [±31.12]	119.09 [±29.54]	100.02 [±28.68]	**<0.001**	105.57 [±29.50]	111.91 [±29.96]	99.79 [±27.90]	**<0.001**	−0.40

Values are means [± standard deviations], median [25th-75th percentile]a or n (%)

STEMI = ST-segment Elevated Myocardial Infarction; NSTE-ACS = Non-ST-segment Elevated Acute Coronary Syndrome; COPD = Chronic Obstructive Pulmonary Disease; MI = Myocardial Infarction; PCI = Percutaneous Coronary Intervention; VF/VT = Ventricular Fibrillation/Ventricular Tachycardia.

**Table 2:** Procedural Characteristics.

Variable	**Total**	**STEMI**	**NSTE-ACS**	**P-value**	**Total**	**STEMI**	**NSTE-ACS**	**P-value**	**Standardized mean difference**
	**(n=1255)**	**(n=1006)**	**(n=249)**		**(n=468)**	**(n=234)**	**(n=234)**		
Time to Procedure (hours)	3.2	2.7	18.3	**<0.001**	5.0	2.3	18.7	**<0.001**	1.7
[25^th^-75^th^]	[1.8–8.1]^a^	[1.8–4.9]^a^	[10.3–27.1]^a^		[2.1–18.4]^a^	[1.6–4.4]^a^	[10.3–27.5]^a^		
Access Site				0.050				<0.001	
Radial	1136/1255 (90.5)	902/1006 (89.7)	234/249 (94.0)		421/468 (90.0)	198/234 (84.6)	223/234 (95.3)		0.27
Femoral	119/1255 (9.5)	104/1006 (10.3)	15/249 (6.0)		47/468 (10.0)	36/234 (15.4)	11/234 (4.7)		−0.27
Catheter Size									
5FR	16/1255 (1.3)	14/1006 (1.4)	2/249 (0.8)	0.67	4/468 (0.9)	2/234 (0.9)	2/234 (0.9)	1.00	0.0
6FR	1234/1255 (98.1)	988/1006 (98.2)	243/249 (97.6)	0.70	460/468 (98.3)	232/234 (99.1)	228/234 (97.4)	0.29	−0.18
7FR	7/1255 (0.6)	3/1006 (0.3)	4/249 (1.6)	**0.045**	4/468 (0.9)	0/234 (0.0)	4/234 (1.7)	0.13	–
8FR	1/1255 (0.1)	1/1006 (0.1)	0/249 (0.0)	1.00	0/468 (0.0)	0/234 (0.0)	0/234 (0.0)	–	–
Culprit									
RCA	516/1255 (41.1)	436/1006 (43.3)	80/249 (32.1)	**0.002**	170/468 (36.3)	92/234 (39.3)	78/234 (33.3)	0.21	−0.12
LM	4/1255 (0.3)	3/1006 (0.3)	1/249 (0.4)	1.00	2/468 (0.4)	1/234 (0.4)	1/234 (0.4)	1.00	0.0
LAD/D	476/1255 (37.9)	406/1006 (40.4)	70/249 (28.1)	**<0.001**	159/468 (34.0)	95/234 (40.6)	64/234 (27.4)	**0.003**	−0.27
CX/IM	259/1255 (20.6)	161/1006 (16.0)	98/249 (39.4)	**<0.001**	137/468 (29.3)	46/234 (19.7)	91/234 (38.9)	**<0.001**	0.48
Culprit Lesion Classification									
A	66/1255 (5.3)	54/1006 (5.4)	12/249 (4.8)	0.85	20/468 (4.3)	8/234 (3.4)	12/234 (5.1)	0.49	0.09
B1	190/1255 (15.1)	159/1006 (15.8)	31/249 (12.4)	0.22	68/468 (14.5)	39/234 (16.7)	29/234 (12.4)	0.24	−0.12
B2	255/1255 (20.3)	211/1006 (21.0)	44/249 (17.7)	0.28	105/468 (22.4)	65/234 (27.8)	40/234 (17.1)	**0.008**	−0.24
C	744/1255 (59.3)	582/1006 (57.9)	162/249 (65.1)	**0.045**	275/468 (58.8)	122/234 (52.1)	153/234 (65.4)	**0.005**	0.27
Multivessel Disease	538/1255 (42.9)	413/1006 (41.1)	125/249 (50.2)	**0.011**	183/468 (39.1)	67/234 (28.6)	116/234 (49.6)	**<0.001**	0.46
Collaterals	275/1255 (21.9)	167/1006 (16.6)	108/249 (43.4)	**<0.001**	140/468 (29.9)	38/234 (16.2)	102/234 (43.6)	**<0.001**	0.74
RENTROP									
1	145/1255 (11.6)	105/1006 (10.4)	40/249 (16.1)	**0.017**	61/468 (13.0)	24/234 (10.3)	37/234 (15.8)	0.10	0.06
2	90/1255 (7.2)	53/1006 (5.3)	37/249 (14.9)	**<0.001**	49/468 (10.5)	13/234 (5.6)	36/234 (15.4)	**0.001**	0.43
3	40/1255 (3.2)	9/1006 (0.9)	31/249 (12.4)	**<0.001**	30/468 (6.4)	1/234 (0.4)	29/234 (12.4)	**<0.001**	1.85
Final TIMI flow									
0	21/1255 (1.7)	16/1006 (1.6)	5/249 (2.0)	0.85	9/468 (1.9)	4/234 (1.7)	5/234 (2.1)	1.00	0.03
1	21/1255 (1.7)	18/1006 (1.8)	3/249 (1.2)	0.71	6/468 (1.3)	4/234 (1.7)	2/234 (0.9)	0.68	−0.06
2	77/1255 (6.1)	62/1006 (6.1)	15/249 (6.0)	1.00	30/468 (6.4)	15/234 (6.4)	15/234 (6.4)	1.00	0.0
3	1136/1255 (90.5)	910/1006 (90.5)	226/249 (90.8)	0.98	423/468 (90.4)	211/234 (90.2)	212/234 (90.6)	1.00	0.01

Values are means [± standard deviations], median [25^th^-75^th^ percentile]^a^ or n (%).

FR = French; RCA = Right Coronary Artery; LM = Left Main artery; LAD/D = Left Anterior Descending artery / Diagonal artery; CX/IM = Circumflex artery / Intermediate artery.

Other abbreviations as in Table 1.

**Table 3:** Procedural Characteristics.

Variable	**Total**	**STEMI**	**NSTE-ACS**	**P-value**	**Total**	**STEMI**	**NSTE-ACS**	**P-value**	**Standardized mean difference**
	**(n=1255)**	**(n=1006)**	**(n=249)**		**(n=468)**	**(n=234)**	**(n=234)**		
Culprit Procedure method									
Thrombus Aspiration	547/1255 (43.6)	475/1006 (47.2)	72/249 (28.9)	**<0.001**	220/468 (47.0)	152/234 (65.0)	68/234 (29.1)	**<0.001**	−0.75
Predilatation	849/1255 (67.6)	657/1006 (65.3)	192/249 (77.1)	**<0.001**	338/468 (72.2)	157/234 (67.1)	181/234 (77.4)	**0.018**	0.22
Postdilatation	572/1255 (45.6)	452/1006 (44.9)	120/249 (48.2)	0.39	215/468 (45.9)	101/234 (43.2)	114/234 (48.7)	0.27	0.11
Culprit									
Number of Stents	1 [1–2]^a^	1 [1–2]^a^	1 [1–2]^a^	0.69	1 [1–2]^a^	1 [1–2]^a^	1 [1–2]^a^	0.10	0.19
Stent Length	26 [18–40]^a^	26 [18–40]^a^	26 [16–44]^a^	0.97	26 [16–40]^a^	24 [18–38]^a^	26 [16–44]^a^	0.19	0.23
Total									
Number of Stents	2 [1–2]^a^	1 [1–2]^a^	2 [1–3]^a^	**0.013**	1 [1–2]^a^	1 [1–2]^a^	2 [1–3]^a^	**<0.001**	0.45
Stent Length	32 [18–56]^a^	31 [18–53]^a^	38 [20–64]^a^	**0.034**	32 [18–56]^a^	28 [18–44]^a^	39 [20–65]^a^	**<0.001**	0.53
Multivessel PCI	364/1255 (29.0)	275/1006 (27.3)	89/249 (35.7)	**0.011**	126/468 (26.9)	43/234 (18.4)	83/234 (35.5)	**<0.001**	0.44
RCA									
Significant	117/1255 (9.3)	88/1006 (8.7)	29/249 (11.6)	0.20	45/468 (9.6)	18/234 (7.7)	27/234 (11.5)	0.21	0.14
Thrombotic	516/1255 (41.1)	436/1006 (43.3)	80/249 (32.1)	**0.002**	170/468 (36.3)	92/234 (39.3)	78/234 (33.3)	0.21	–0.12
CTO	66/1255 (5.3)	49/1006 (4.9)	17/249 (6.8)	0.28	24/468 (5.1)	8/234 (3.4)	16/234 (6.8)	0.14	0.17
Collaterals	221/1258 (17.6)	155/1009 (15.4)	66/249 (26.5)	**<0.001**	95/468 (20.3)	31/234 (13.2)	64/234 (27.4)	**<0.001**	0.42
LM									
Significant	39/1255 (3.1)	30/1006 (3.0)	9/249 (3.6)	0.76	9/468 (1.9)	1/234 (0.4)	8/234 (3.4)	**0.043**	0.46
Thrombotic	4/1255 (0.3)	3/1006 (0.3)	1/249 (0.4)	1.00	2/468 (0.4)	1/234 (0.4)	1/234 (0.4)	1.00	0.0
CTO	0/1255 (0.0)	0/1006 (0.0)	0/249 (0.0)	–	0/468 (0.0)	0/234 (0.0)	0/234 (0.0)	–	–
Collaterals	0/1255 (0.0)	0/1006 (0.0)	0/249 (0.0)	–	0/468 (0.0)	0/234 (0.0)	0/234 (0.0)	–	–
LAD/D									
Significant	251/1255 (20.0)	188/1006 (18.7)	63/249 (25.3)	**0.025**	90/468 (19.2)	30/234 (12.8)	60/234 (25.6)	**0.001**	0.38
Thrombotic	476/1255 (37.9)	406/1006 (40.4)	70/249 (28.1)	**<0.001**	159/468 (34.0)	95/234 (40.6)	64/234 (27.4)	**0.003**	−0.27
CTO	32/1255 (2.5)	23/1006 (2.3)	9/249 (3.6)	0.33	12/468 (2.6)	5/234 (2.1)	7/234 (3.0)	0.77	0.06
Collaterals	107/1255 (8.5)	64/1006 (6.4)	43/249 (17.3)	**<0.001**	55/468 (11.8)	17/234 (7.3)	38/234 (16.2)	**0.004**	0.34
CX/IM									
Significant	202/1255 (16.1)	163/1006 (16.2)	39/249 (15.7)	0.91	57/468 (12.2)	20/234 (8.5)	37/234 (15.8)	**0.024**	0.26
Thrombotic	259/1255 (20.6)	161/1006 (16.0)	98/249 (39.4)	**<0.001**	137/468 (29.3)	46/234 (19.7)	91/234 (38.9)	**<0.001**	0.48
CTO	34/1255 (2.7)	29/1006 (2.9)	5/249 (2.0)	0.59	10/468 (2.1)	5/234 (2.1)	5/234 (2.1)	1.00	0.0
Collaterals	59/1255 (4.7)	31/1006 (3.1)	28/249 (11.2)	**<0.001**	33/468 (7.1)	7/234 (3.0)	26/234 (11.1)	**0.001**	0.47
Complication	107/1255 (8.5)	93/1006 (9.2)	14/249 (5.6)	0.09	40/468 (8.5)	26/234 (11.1)	14/234 (6.0)	0.07	−0.16
No Reflow	79/1255 (6.3)	70/1006 (7.0)	9/249 (3.6)	0.07	31/468 (6.6)	22/234 (9.4)	9/234 (3.8)	**0.026**	−0.19
Dissection	14/1255 (1.1)	12/1006 (1.2)	2/249 (0.8)	0.85	5/468 (1.1)	3/234 (1.3)	2/234 (0.9)	1.00	−0.0
Perforation	11/1255 (0.9)	8/1006 (0.8)	3/249 (1.2)	0.81	4/468 (0.9)	1/234 (0.4)	3/234 (1.3)	0.62	0.14
Side Branch Occlusion	3/1255(0.2)	3/1006 (0.3)	0/249 (0.2)	0.89	0/468 (0.0)	0/234 (0.0)	0/234 (0.0)	–	–
Medication at Discharge									
Aspirin	1237/1256 (98.6)	990/1006 (98.4)	247/249 (99.2)	0.52	463/468 (98.9)	230/234 (98.3)	233/234 (99.6)	0.37	0.10
Clopidogrel	101/1255 (8.0)	67/1006 (6.7)	34/249 (13.7)	**<0.001**	50/468 (10.7)	18/234 (7.7)	32/234 (13.7)	0.052	0.22
Prasugrel	182/1255 (14.5)	175/1006 (17.4)	7/249 (2.8)	**<0.001**	13/368 (2.8)	7/234 (3.0)	6/234 (2.6)	1.00	−0.02
Ticagrelor	960/1255 (76.5)	755/1006 (75.0)	205/249 (82.3)	**0.019**	400/468 (85.5)	207/234 (88.5)	193/234 (82.5)	0.09	−0.19
NOAC	16/1255 (1.3)	11/1006 (1.1)	5/249 (2.0)	0.40	5/468 (1.1)	0/234 (0.0)	5/234 (2.1)	0.072	–

## 3 Results

### 3.1 Baseline characteristics

For the total population, the patients’ baseline characteristics are shown in Table 1. The prevalence of hypertension and hypercholesterolemia were significantly higher in the NSTE-ACS group. In addition, history of a previous MI or PCI was higher in the NSTE-ACS group in the total population.

Further, the GRACE scores are significantly higher in the STEMI group.

After matching, the prevalence of hypertension and hypercholesterolemia remain significantly higher in the NSTE-ACS group (standardized mean difference (SMD) 0.24 and 0.23 respectively). The prevalence of current smokers (i.e. currently or stopped smoking in past year) was significantly higher in the STEMI group in the matched population (SMD −0.21). Also, The GRACE scores in the STEMI group remain higher compared to the NSTE-ACS group (SMD −0.40). Other cardiovascular risk factors were highly prevalent in both groups, but did not differ significantly between the two groups.

### 3.2 Procedural characteristics

In the total population, the time from onset of symptoms to start of PCI was significantly longer in NSTE-ACS patients compared to STEMI patients, median 18.3 h [10.3–27.1] vs 2.7 [1.8–4.9] hours with p < 0.001. In NSTE-ACS patients, the culprit vessel was significantly more often the circumflex (Cx) artery (39.4 % in NSTE-ACS patients vs 16.0 % in STEMI patients; p < 0.001). Coronary collaterals occurred more frequently in the NSTE-ACS group than in the STEMI group, 43.4 % vs 16.6 % (p < 0.001). Multivessel PCI was more frequently performed in the NSTE-ACS group in comparison to the STEMI group (35.7 % vs 27.3 %; p = 0.011).

In the matched population, the Cx remained to be significantly more often the culprit in the NSTE-ACS group. Secondly, collaterals still occur more often in NSTE-ACS patients, including RENTROP 2/3 collaterals. Combined B2/C lesion classification was comparable between the two groups (82.5 % in NSTE-ACS group vs 79.9 % in STEMI group). Finally, multivessel PCI, also reflected in higher total number of stents and higher total stent length used during PCI, was significantly higher in the NSTE-ACS patients (p < 0.001).

### 3.3 Outcome

**Fig. 2:**
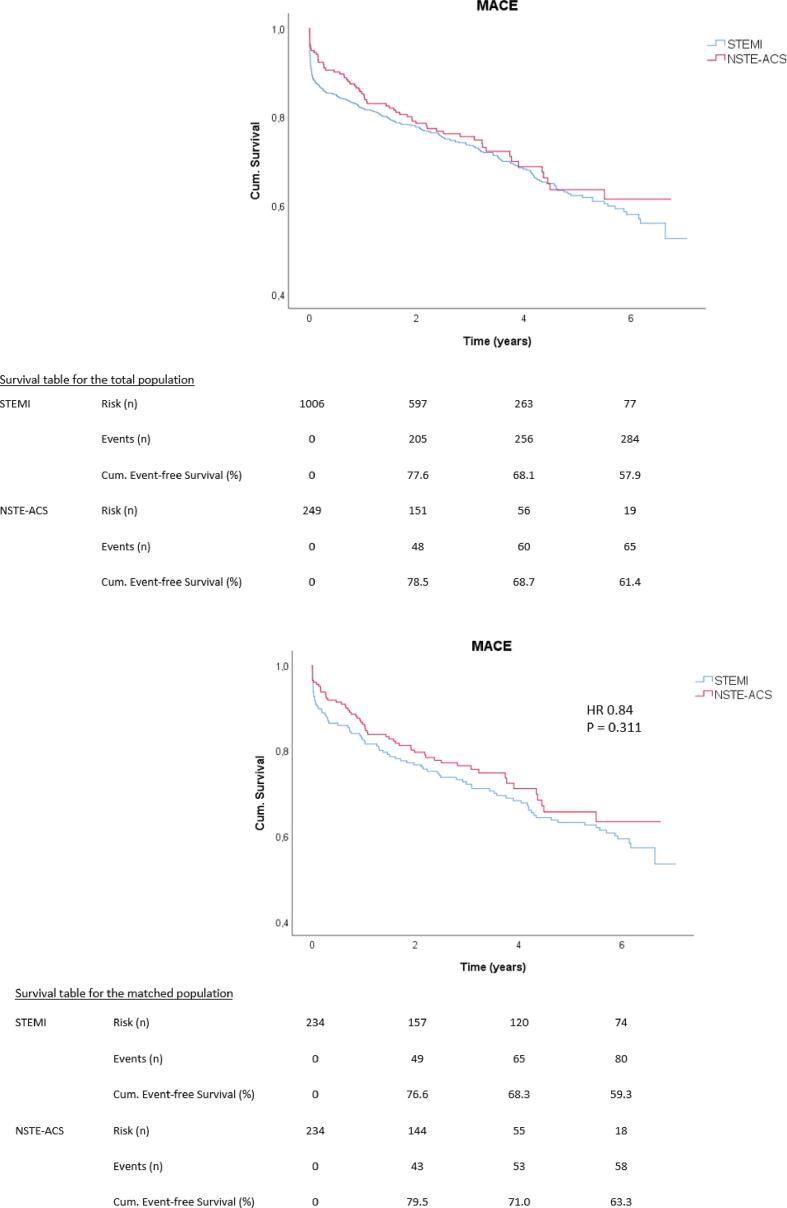
MACE rates and survival tables for the total population (A) and matched population (B).

Table 4 shows the number of events for both the primary and secondary outcomes In the total population, the median follow-up time was 2.6 [1.3–4.4] years. In the NSTE-ACS group, the median follow-up time was 2.9 [1.5–4.4] years vs 2.7 [1.2–4.4] years in the STEMI group (p = 0.63). At the median follow-up time, the cumulative event-free survival was 76.1 % in the NSTE-ACS group and 74.6 % in the STEMI group. During follow-up, there was no significant difference in MACE rates between the NSTE-ACS and the STEMI group (cumulative event-free survival at 6 year follow-up 61.4 % vs 57.9 %, log-rank test p = 0.42). The Kaplan-Meier curves for MACE in the total population and in the matched population are shown in Fig. 2.

**Table 4:** Primary and Secondary Outcomes.

Outcomes	**Total**	**STEMI**	**NSTE-ACS**	**Log-rank test P-value**	**Total**	**STEMI**	**NSTE-ACS**	**HR [95% CI]**	**P-value**
	**(n=1255)**	**(n=1006)**	**(n=249)**		**(n=468)**	**(n=234)**	**(n=234)**		
Primary Outcome	352/1255 (28.0)	287/1006 (28.5)	65/249 (26.1)	0.42	141/468 (30.1)	83/234 (35.5)	58/234 (24.8)	0.87 [0.60–1.18]	0.31
MACE									
Secondary Outcome									
MI	81/1255 (6.5)	60/1006 (6.0)	21/249 (8.4)	0.19	34/468 (7.3)	16/234 (6.8)	18/234 (7.7)	1.50 [0.74–3.03]	0.26
Stroke	35/1255 (2.8)	31/1006 (3.1)	4/249 (1.6)	0.20	12/468 (2.6)	9/234 (3.8)	3/234 (1.3)	0.53 [0.14–2.09]	0.37
CABG	17/1255 (1.4)	14/1006 (1.4)	3/249 (1.2)	0.79	9/468 (1.9)	6/234 (2.6)	3/234 (1.3)	0.67 [0.16–2.78]	0.58
UTVR	69/1255 (5.5)	58/1006 (5.8)	11/249 (4.4)	0.36	28/468 (6.0)	18/234 (7.7)	10/234 (4.3)	0.62 [0.30–1.30]	0.21
Death (all-cause)	210/1255 (16.7)	170/1006 (16.9)	40/249 (16.1)	0.67	84/468 (17.9)	48/234 (20.5)	36/234 (15.4)	0.85 [0.54–1.34]	0.49

For the matched population, the median follow-up time was 3.2 years [1.4–6.0] (i.e. 2.9 [1.5–4.4] years in the NSTE-ACS and 5.6 [0.7–6.2] years in the STEMI group (p < 0.001)). At median follow-up, the cumulative event-free survival was 75.6 % in the NSTE-ACS group vs 71.0 % in the STEMI group. At 6 year follow-up, the number of patients still at risk in the NSTE-ACS group was smaller than in the STEMI group. The MACE rate did not differ between the two groups (HR = 0.84 [95 % CI 0.60 – 1.12] with p = 0.33). As shown in Table 4, also for the individual components of MACE, no difference was observed.

## 4 Discussion

The main finding of the study is that NSTE-ACS patients with a totally occluded culprit vessel have similar clinical outcomes as compared to STEMI patients with an occluded culprit vessel.

In STEMI patients with baseline TIMI 0 flow, rapid recanalization is mandatory to improve outcome. During our study period, early coronary angiography (within 24 h of hospital admission) only had to be performed in high-risk NSTE-ACS patients [11]. Therefore, NSTE-ACS patients with a totally occluded coronary artery vessel but without these high risk features might be excluded from early emergent coronary angiography. A recent meta-analysis by Hung et al. reported that NSTEMI patients with TIMI 0/1 flow have increased mortality rates and re-infarction rates compared to NSTEMI patients without a totally occluded culprit [20]. Moreover, Karwowski et al. found that NSTEMI patients with a total occlusion of the Cx had a higher in-hospital (30 days) mortality than non-total occlusions of the Cx [21]. Bearing this in mind, NSTE-ACS patients with TIMI 0 flow might benefit from rapid recanalization like in STEMI patients.

The time from symptom onset to the start of the procedure was significantly longer in NSTE-ACS patients compared to STEMI patients, consistent with previous research [21], [22], [23]. However, the effect of timing of PCI on NSTE-ACS patients specifically with TIMI 0 flow remains unclear, as some studies have found inconclusive results on the benefit of rapid versus delayed reperfusion in NSTEMI patients [12], [13]. Better identification of patients with TIMI 0 flow could aid in future studies clarify the optimal timing of recanalization in NSTE-ACS patients with TIMI 0 flow.

Although TIMI 0 flow at baseline is a negative prognostic factor and time to recanalization is significantly longer in NSTE-ACS patients, this was not associated with inferior outcomes compared to STEMI patients. These findings might be explained by several factors. Firstly, this study shows that the left circumflex artery is significantly more often the culprit in NSTE-ACS patients compared to STEMI patients. The sensitivity of the 12-lead electrocardiogram is lower in detecting occlusions in the Cx, most likely because of its posterolateral location and the lack of corresponding leads [3], [24]. Consequently, as shown in numerous studies, the Cx is more frequently the culprit in NSTEMI patients with a totally occluded vessel [9], [20], [21], [22], [25]. The Cx generally supplies a relatively smaller area compared to the other two main coronary arteries [9]. Therefore, a myocardial infarction caused by total occlusion of the Cx usually leads to a smaller infarction size. In addition, Karwowski et al. reports that the infarction sizes of NSTEMI’s with TIMI 0 flow are smaller in comparison to STEMI’s with a total occlusion [21].

There were significantly more collaterals present in the NSTE-ACS group compared to the STEMI group in this study. This may be due to the longer duration of ischemic symptoms to angiography in the NSTE-ACS group, as collaterals are known to be better developed in patients with longer symptom duration [26]. However, it is also possible that these collaterals were present before the total occlusion of the vessel, which could explain the lack of ST segment elevation and smaller infarct size in NSTE-ACS patients with completely occluded vessels. While the potential benefits of collaterals in ACS patients are still unclear, with some studies finding inconclusive results [4], [27], [28], Elsman et al. show that RENTROP 2/3 collaterals may have a protective effect [29]. Further research is needed to fully understand the role of collaterals in ACS patients with TIMI 0 flow.

In both the total and the matched cohort there is more multivessel disease present in the NSTE-ACS group. It is known that multivessel disease in ACS is associated with increased mortality rates [27], [28], [30]. However, most of these studies are outdated with only limited percentage of complete revascularization. Although not so compelling as in STEMI patients, complete revascularization seems also beneficial in NSTEMI patients when compared to culprit only revascularization [28], [31], [32]. In the NSTE-ACS group, multivessel PCI is performed more often in both the total and the matched cohort.

Coronary dominance and the target vessel diameter could additionally be of influence on the outcomes. Although the coronary dominance was not registered in our study, Chua et al. [33] showed that ACS patients with an occlusion of the Cx and right or mixed coronary dominance were less likely to occur with ST-segment changes on the ECG. Furthermore, it is hypothesized that right coronary artery dominance could have a protective role in ACS patients with a total occlusion of the Cx due to several pathways [24]. This way, it could be that a substantial portion of our NSTE-ACS group with Cx occlusion benefitted from its coronary dominance. Besides, Asselbergs et al. [34] reported that vessel size is predictive for cardiovascular events. Thus, a culprit small in diameter is associated with a smaller infarction size [35].

There were some differences in antiplatelet therapy in the total cohort. During the study period, the preferred P2Y12 receptor inhibitor for ACS patients in our center was ticagrelor. However, due to the start of a medication study, STEMI patients were treated with prasugrel from November 2017 when they were brought in directly with the ambulance [36]. Furthermore, a significant amount of patients was also treated with clopidogrel, especially in NSTE-ACS patients and more than one would expect compared to NOAC use. As this is a retrospective study and the choice of antiplatelet therapy was per physician’s discretion, we have no explanation for this high percentage of clopidogrel prescription.

### 4.1 Limitations

This study has several limitations. Firstly, we have no information on infarct size or left ventricular ejection fraction after the ACS. Secondly, after PS matching, some prognostic factors (e.g. Kilip class and GRACE score) still differed significantly. Thirdly, this is a retrospective study and therefore more likely to be influenced by confounding bias. To minimize this, propensity score matching was performed and the analyses were repeated with the matched population.

## 5 Conclusion

This study shows that NSTE-ACS patients with TIMI 0 flow have significant longer onset of symptoms to PCI than STEMI patients. NSTE-ACS patients with TIMI 0 flow have similar clinical outcomes in comparison to STEMI patients, which would suggest that the difference in timing in coronary angiography in the current guidelines for STEMI and NSTE-ACS are justified. However, due to missing information on infarct size and left ejection fraction and remaining differences in some prognostic variables even after PS matching, further research is warranted to determine the optimal timing of PCI in NSTE-ACS patients with TIMI 0 flow.

## Declaration of Competing Interest

The authors declare that they have no known competing financial interests or personal relationships that could have appeared to influence the work reported in this paper.
